# Lack of Hfe and TfR2 in Macrophages Impairs Iron Metabolism in the Spleen and the Bone Marrow

**DOI:** 10.3390/ijms25179142

**Published:** 2024-08-23

**Authors:** Stefano Comità, Patrizia Falco, Mariarosa Mezzanotte, Maja Vujić Spasić, Antonella Roetto

**Affiliations:** 1 Department of Clinical and Biological Sciences, University of Turin, 10126 Turin, Italy; stefano.comita@unito.it (S.C.); patrizia.falco94@gmail.com (P.F.); 2 Department of Neuroscience Rita Levi Montalcini, Neuroscience Institute Cavalieri Ottolenghi, University of Turin, 10126 Turin, Italy; mariarosa.mezzanotte@unito.it; 3 Institute of Comparative Molecular Endocrinology, Ulm University, Helmholtzstr. 8/1, 89081 Ulm, Germany; maja.vujic@uni-ulm.de

**Keywords:** iron, macrophages, animal model, hemochromatosis

## Abstract

Iron is a vital element involved in a plethora of metabolic activities. Mammalian systemic iron homeostasis is mainly modulated by hepcidin, the synthesis of which is regulated by a number of proteins, including the hemochromatosis-associated proteins Hfe and Transferrin Receptor 2 (TfR2). Macrophages play versatile functions in iron homeostasis by storing iron derived from the catabolism of erythrocytes and supplying iron required for erythropoiesis. The absence of Hfe in macrophages causes a mild iron deficiency in aged mice and leads to an overproduction of the iron exporter Ferroportin 1 (Fpn1). Conversely, *TfR2* gene silencing in macrophages does not influence systemic iron metabolism but decreases transcription of the macrophage Fpn1 in adult mice and modulates their immune response. This study investigated cellular and systemic iron metabolism in adult and aged male mice with macrophage-specific Hfe and TfR2 silencing (double knock-out, DKO). Serum iron parameters were significantly modified in aged animals, and significant differences were found in hepatic hepcidin transcription at both ages. Interestingly, splenic iron content was low in adult DKOs and splenic Fpn1 transcription was significantly increased in DKO animals at both ages, while the protein amount does not reflect the transcriptional trend. Additionally, DKO macrophages were isolated from mice bone marrow (BMDMs) and showed significant variations in the transcription of iron genes and protein amounts in targeted mice compared to controls. Specifically, Tranferrin Receptor 1 (TfR1) increased in DKO adult mice BMDMs, while the opposite is observed in the cells of aged DKO mice. *Fpn1* transcript was significantly decreased in the BMDMs of adult DKO mice, while the protein was reduced at both ages. Lastly, a significant increase in Erythropoietin production was evidenced in aged DKO mice. Overall, our study reveals that Hfe and TfR2 in macrophages regulate hepatic Hepc production and affect iron homeostasis in the spleen and BMDMs, leading to an iron deficiency in aged animals that impairs their erythropoiesis.

## 1. Introduction

Iron is an essential element required for the proper functioning of living organisms. In mammals, iron is primarily obtained through the absorption of dietary inorganic non-heme and heme iron through specific pathways (reviewed in [1,2]). Systemic iron availability is regulated by the iron hormone hepcidin (Hepc), which is produced by hepatocytes and controls the transfer of iron from enterocytes, macrophages, and hepatocytes to plasma [3]. Specifically, Hepc binds the iron exporter Ferroportin 1 (Fpn1), triggering Fpn1 internalization and its proteolysis and reducing de facto iron export from cells [4,5]. 

*Hepc* transcription is controlled by numerous upstream regulators [3,6,7,8,9], including hemochromatosis-associated proteins such as Hfe and TfR2. Mutations in human *HFE* and *TFR2* genes cause Hemochromatosis type 1 and 3, respectively, a genetic heterogeneous disease that results in body iron accumulation due to abnormally low Hepc production [10,11]. Consequently, patients present with hyperferremia, high transferrin saturation (TS), and accumulation of iron in parenchymal tissues, particularly in the liver and the heart [12]. The same phenotype has been successfully recapitulated in animal models of hereditary hemochromatosis [13]. According to the most recent functional model, Hfe alternatively binds transferrin receptors 1 and 2 (TfR1 and TfR2, respectively) in its extracellular α1-α2 domain and is required for the physiological regulation of hepatic synthesis of Hepc [14]. Accordingly, mice with constitutive or liver-specific inactivation of *Hfe* or *TfR2*, or both, showed reduced levels of hepatic Hepc expression and a considerable iron overload in the liver, which was the highest in mice with combined Hfe and TfR2 deficiencies [15,16,17,18,19,20,21,22,23,24]. These latter observations suggested that the functions of Hfe and TfR2 are not entirely co-dependent, even though they are both involved in the regulation of hepatic Hepc.

In addition to the essential role of Hfe and TfR2 in hepatic Hepc regulation, abnormal iron metabolism in myeloid cells/macrophages has been documented in patients with hereditary hemochromatosis. For example, patients with mutations in *HFE* or *TFR2* have iron-deficient splenic macrophages despite a body iron overload [25]. Moreover, the TfR2 beta isoform is highly expressed in macrophages and its inactivation in mice results in iron perturbation in splenic macrophages and the bone marrow [23,26]. In addition to pathological conditions, natural aging influences macrophages and their ability to maintain iron homeostasis [25,27].

Macrophages are cells present in all tissues and, among their different metabolic functions, they are responsible for handling iron, thereby regulating the tissue iron pool and the subsequent tissue microenvironment [27]. Iron is redistributed by macrophages for two primary reasons: it must be supplied for cellular needs, being essential for erythropoiesis, and it is sequestered for bacteriostasis. However, tissue-resident macrophages may also serve as a bioavailable iron storage compartment and be used according to local tissue needs, thus local iron availability must be controlled to ensure cellular iron homeostasis. Macrophages take up transferrin (Tf)-bound iron through the well-known holo-Tf/TfR1 pathway, while the unbound iron is introduced into the cells via a main transmembrane channel (Divalent Metal Channel, DMT1) [27]. Additionally, macrophages import iron in heme and hemoglobin derived from intravascular RBC hemolysis through Heme-hemopexin (Hx-Heme) and haemoglobin-haptoglobin (Hb-Hp)-mediated endocytosis, respectively [2]. Excess iron that is not utilized is either exported via Fpn1 or stored within the cytosol in a stable form within ferritin (Ft). This ability of macrophages to regulate iron availability in the microenvironment, thereby contributing to cellular/tissue function, depends on local iron necessity and may be independent of systemic iron homeostasis [27]. Specifically, iron-rich macrophages within erythroid islands in the bone marrow are suggested to provide iron for erythropoiesis and express many proteins involved in iron metabolism, such as TfR1, Fpn1, and Hepc [28]. Conversely, splenic red pulp macrophages play a role in recycling iron from senescent erythrocytes [29]. Finally, studies performed in aged mice carrying a targeted deletion of *Hfe* in their myeloid cells (HfeLysMCre mice) revealed a noteworthy variation in hepatic and splenic iron parameters and impaired iron metabolism in isolated macrophages independent of *Hepc* expression [30]. 

To determine if the Hfe and Tfr2 proteins in macrophages influence iron metabolism separately, collectively, or at different ages, a novel murine mouse line was developed that contained double *Hfe*/*TfR2* silencing in these cell types (Hfe/TfR2 KO, DKO). Herein, the repercussions of lacking macrophage Hfe/TfR2 activities on systemic and cellular iron metabolism are described.

Moreover, DKO animals were investigated at the same ages in which macrophage *Hfe* or *TfR2* single KOs displayed improper iron metabolism.

Our data show the indispensable role of Hfe and TfR2 in the regulation of hepatic hepcidin production and in iron homeostasis in the spleen and bone marrow-derived macrophages (BMDMs), which are crucial for iron management during aging.

## 2. Results

Hfe/TfR2 floxed mice were obtained by crossing mice carrying the floxed *Hfe* allele [31] (from a C57BL/6J genetic background) with mice carrying the floxed *TfR2* allele, named Tfr2 KI in the paper by Roetto et al. [23] (from a sv129 genetic background). These mice were bred with LysMCre (+) transgenic mice (from a C57BL/6 genetic background), expressing Cre recombinase under the control of the mouse lysozyme M promoter (LysMCre (+)), to obtain Hfe floxed-TfR2 floxed LysMCre (+) (Hfe/TfR2 double knock out, DKO) and Hfe/TfR2 Hfe floxed-TfR2 floxed LysMCre (-) (controls, CTRLs) mice. Transcription of both *Hfe* and *Tfr2* genes resulted significantly reduced in Bone Marrow-Derived Macrophages (BMDMs) of DKO mice (*p* = 0.0309 and *p* = 0.0433) (Figure S1). No evident changes from the mendelian ratio in newborn genotypes nor in pups viability have been observed. 

### 2.1. Lack of Hfe/TfR2 Affects Systemic Iron Levels in DKO Mice 

**Figure 1:**
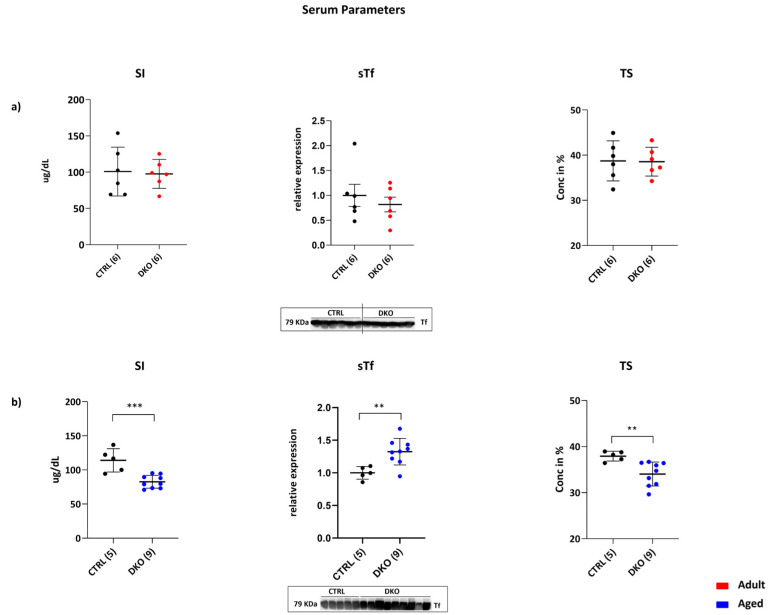
DKO mice serum parameters. (**a**) Adult DKO Serum Iron (SI), Serum Transferrin (sTS), and Transferrin Saturation (TS) were comparable to those of age-matched CTRLs. (**b**) Aged DKO mice SI and TS levels were significantly lower vs. those of age-matched CTRLs. On the contrary, sTf levels were significantly increased vs. CTRLs. Tf was quantified through Western blot analysis of the protein in freshly isolated peripheral blood sera from adult and aged mice. The number of animals analyzed is in parentheses. (** *p* ≤ 0.01; *** *p* ≤ 0.001).

Systemic iron parameters in adult and aged DKO male mice (10 weeks and 45–52 weeks of age, respectively) were investigated. The analysis of serum iron (SI), serum transferrin (sTf), and Transferrin Saturation (TS) in adult DKO mice revealed no significant differences vs. the CTRL group (Figure 1a), while in aged DKO mice, SI and TS were significantly decreased compared to age-matched CTRLs (*p* = 0.0007 and *p* = 0.0084, respectively), and sTf showed a relevant increase vs. age-matched controls (*p* = 0.0063) (Figure 1b). These data show that a combined Hfe and TfR2 deficiency in macrophages results in the development of hypoferremia in aged mice.

### 2.2. Lack of Hfe/TfR2 Induces Hepatic Hepc Response but Not Changes in Liver Iron Amount in DKO Mice 

**Figure 2:**
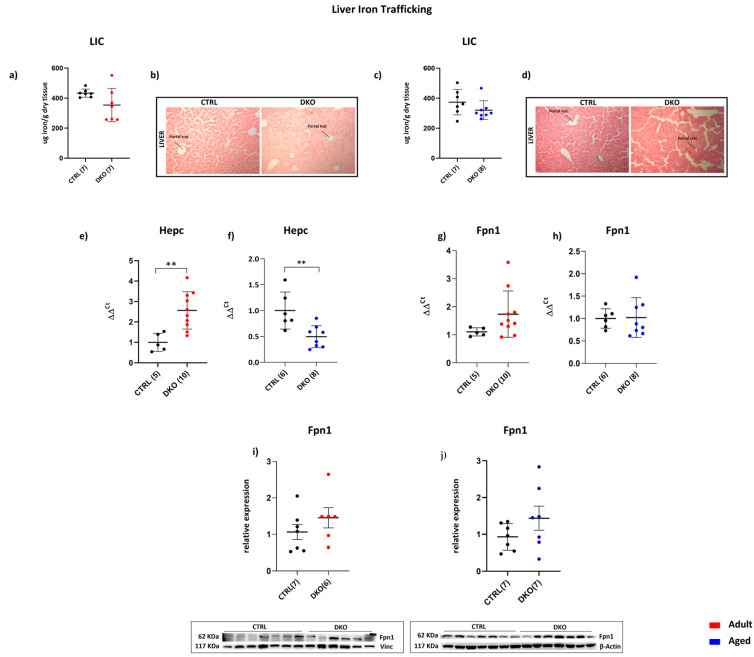
Hepatic iron metabolism of DKO mice. Liver Iron Content (LIC) of adult (**a**) and aged (**c**) DKO mice was not changed. Consistently, Perls’ Prussian Blue on liver tissue confirmed no significant differences in iron deposits in adult (**b**) and aged (**d**) DKO mice vs. age-matched CTRLs. Hepatic Hepc transcription in adult (**e**) and aged (**f**) DKO mice was significantly increased and decreased, respectively, vs. age-matched CTRLs. Fpn1 transcripts (**g**,**h**) and Fpn1 protein amounts (**i**,**j**) remain comparable to those of age-matched CTRLs. 2^-delta delta Ct evaluation was used for transcriptional analyses. The number of analyzed animals in each experiment is in parentheses. (** *p* ≤ 0.01).

The liver iron content (LIC) of adult and aged DKO mice was not changed (Figure 2a,c) compared to age-matched CTRLs. Consistently, staining for iron deposition in the liver showed no evident iron accumulation between all the mice groups at both ages (Figure 2b,d). Therefore, the lack of Hfe and TfR2 in macrophages was not associated with iron amount changes in the liver, differently from the hepatic iron overload that was reported in DKO constitutive mice [24]. On the contrary, hepatic *Hepc* expression was increased in adult DKO animals (*p* = 0.0034) and halved in aged ones (*p* = 0.0062) (Figure 2e,f). Surprisingly, no significant changes could be observed in either hepatic *Fpn1* transcript (Figure 2g,h) or in the protein amount at both animal ages (Figure 2i,j).

These data imply that a combined lack of Hfe and TfR2 in macrophages does not affect liver iron deployment. On the other hand, *Hfe* and *Tfr2* silencing in macrophages induces hepatic Hepc production in adult DKO animals and impacts systemic iron availability in aged mice.

### 2.3. Lack of Hfe/TfR2 Affects Splenic Iron Metabolism in DKO Mice 

**Figure 3:**
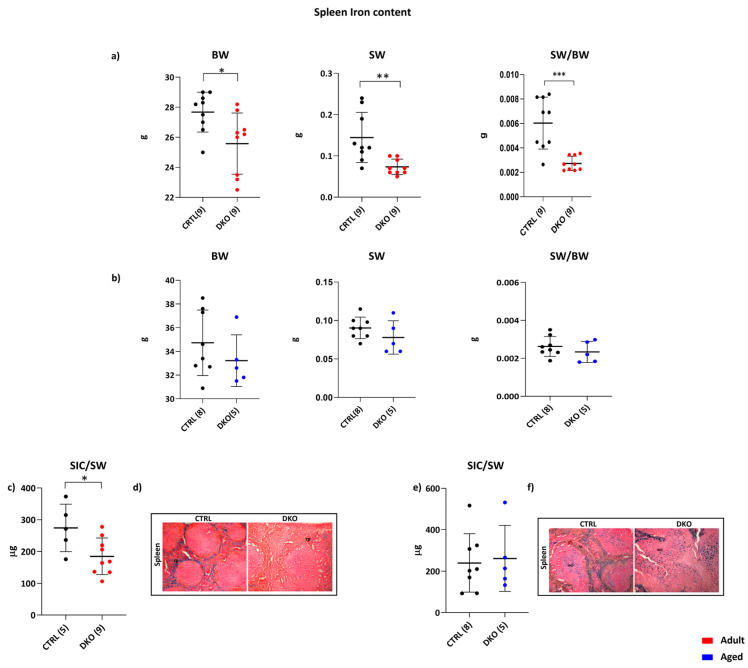

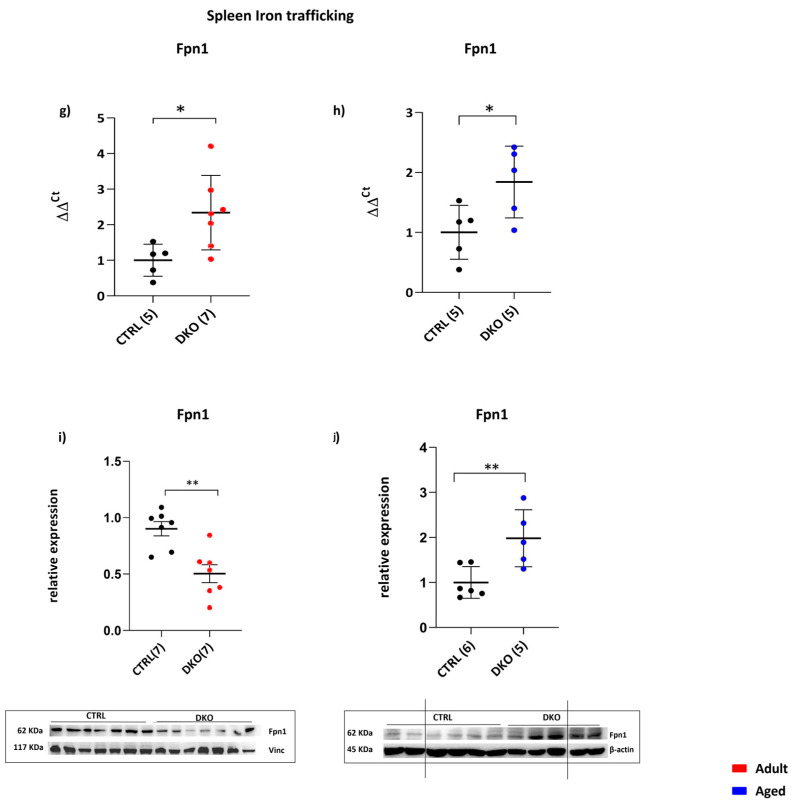
DKO Spleen Iron metabolism. Adult (**a**) and aged (**b**) mice’s body weight (BW), total spleen weight (SW), and SW/BW ratio were significantly decreased only in adult DKO animals vs. age-matched CTRLs. Spleen Iron Content (SIC) was decreased in adult DKO mice (**c**), while no significant variations were observed in aged DKO animals (**e**) vs. age-matched CTRLs. Lower iron levels were confirmed in adult DKO spleens (**d**) while comparable iron deposits were present in aged DKO animals’ spleens (**f**) vs. age-matched CTRLs, all evidenced with Perls’ Prussian blue staining. Images are shown at 10X magnification. rp: red pulp. Splenic Fpn1 mRNA expression is significantly increased in adult (**g**) and aged (**h**) DKO mice vs. age-matched CTRLs. Conversely, the amount of Fpn1 protein is reduced in the spleens of adult DKO animals (**i**) and increased in aged DKO spleens (**j**) vs. age-matched CTRLs. The number of animals analyzed is in parentheses. (* *p* ≤ 0.05; ** *p* ≤ 0.01; *** *p* ≤ 0.001).

In contrast to liver iron content, spleen iron levels (SIC) were significantly affected in adult DKO mice. Interestingly, these mice were lighter than controls (*p* = 0.0196), and the spleen weight (SW) of these mice was halved compared to the spleens of CTRLs (*p* = 0.0041) (Figure 3a). Normalization of SW to the animal’s body weight (BW) revealed a significantly lower ratio compared to adult CTRLs (*p* = 0.004) (Figure 3a). All these parameters were not significantly different in aged DKO vs. CTRLs animals (Figure 3b).

Simultaneously, the splenic iron content normalized to the total spleen weight (SIC/SW) was significantly lower in adult DKO mice vs. CTRLs (*p* = 0.0027) (Figure 3c). This result was confirmed by Perls’ histological analysis, where adult DKO mice showed lower iron deposits in red pulp (Figure 3d). On the contrary, SIC/SW was not significantly changed in aged DKO mice vs. CTRLs (Figure 3e) and the aged animals’ spleens showed no differences in iron amounts vs. aged-matched CTRLs (Figure 3f). 

A statistically significant increase in Fpn1 mRNA in adult and aged DKO mice compared to the respective controls was demonstrated (*p* = 0.0237 and *p* = 0.0365, respectively) (Figure 3g,h). On the contrary, splenic Fpn1 protein was decreased in adult DKO mice (*p* = 0.0020) (Figure 3i) and increased in aged DKO mice compared to age-matched CTRLs (Figure 3j, (*p* = 0.0099). No modification in the splenic transcription of the iron importers TfR1 and DMT1 could be observed in DKO vs. CTRL animals at both ages (Figure S2). While the decrease in the amount of splenic Fpn1 is explainable as a response to increased hepatic Hepcidin production, these data suggest that the lack of Hfe/TfR2 leads to increased *Fpn1* transcript in DKO mice independently from the animals’ age. 

### 2.4. Iron Metabolism in Bone Marrow-Derived Macrophages Is Compromised by the Lack of Hfe/TfR2

**Figure 4:**
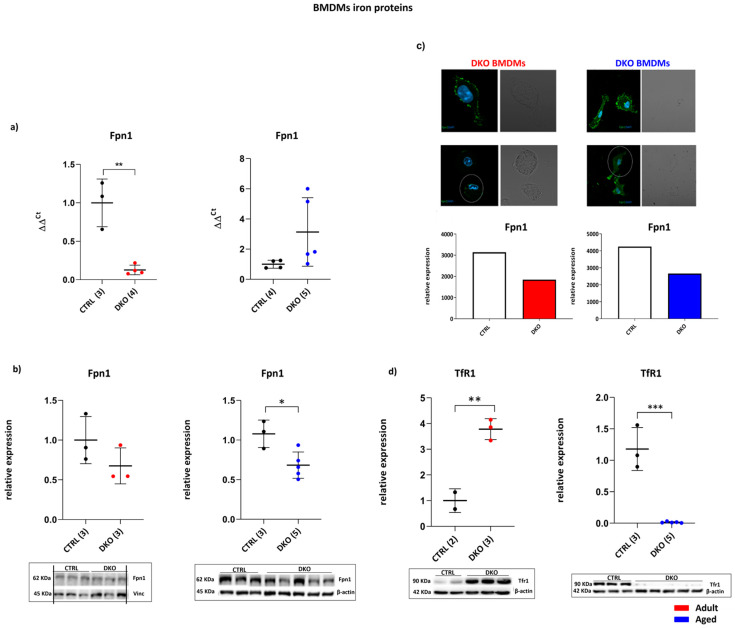
BMDM iron proteins. (**a**) Relative Fpn1 expression was decreased in adult DKO BMDMs while no significant changes were observed in aged animals. (**b**) Fpn1 amounts in adult and aged DKO did not change in adult DKO BMCMs but were significantly reduced in aged DKO mice compared to CTRLs. (**c**) Relative amounts of Fpn1 were reduced both in adult and in aged DKO BMDMs, measured using immunofluorescence. The data are representative of three mice BMDMs/groups normalized to DAPI. Signal strength was measured using ImageJ 1.38v software. Nuclei stained with DAPI are outlined in blue. (**d**) Immunoblot and relative TfR1 protein amount is increased in BMDMs from adult DKO mice and was barely produced in aged DKO mice vs. age-matched CTRLs. The number of animals utilized for gene transcription and protein amounts is in parentheses. (* *p* ≤ 0.05; ** *p* ≤ 0.01; *** *p* ≤ 0.001).

Since macrophages exert an important role in providing iron for erythropoiesis, the effect of Hfe/TfR2 deficiency was analyzed in isolated bone marrow-derived macrophages (BMDMs) from adult and aged DKO mice to evaluate if *Hfe* and *TfR2* silencing could compromise RBC production. *Fpn1* transcripts were considerably reduced in adult mice (*p* = 0.0024) and not significantly changed in aged DKO mice vs. age-matched CTRLs (Figure 4a). Immunoblot analysis showed lower Fpn1 levels in the BMDMs of adult DKO mice and a statistically significant reduction of Fpn1 in aged DKO mice compared to CTRLs (*p* = 0.0183) (Figure 4b). The Fpn1 decrease was confirmed by immunofluorescence analysis (Figure 4c).

To verify if iron entry was modified in these cells, the TfR1 iron transporter was investigated. Significantly increased TfR1 protein levels in the BMDMs of adult DKO mice (*p* = 0.0056) were evident, while they were nearly absent in the BMDMs of aged DKO mice, compared to their respective CTRLs (*p* = 0.0002) (Figure 4d). 

**Figure 5:**
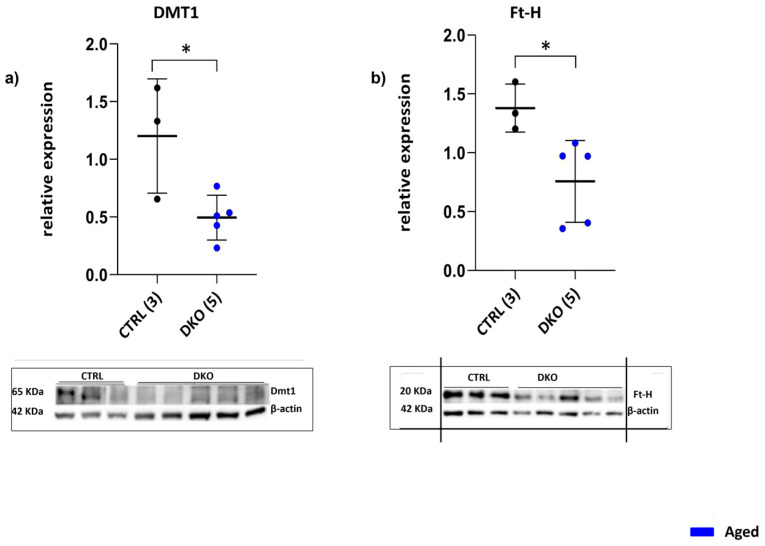
BMDM iron proteins. (**a**) DMT1 protein expression in aged DKO BMDMs was significantly decreased vs. CTRLs. (**b**) Amount of FtH protein was lower than the CTRL in aged DKO mice. The number of analyzed animals is in parentheses. (* *p* ≤ 0.05).

To further investigate iron trafficking and amounts in the BMDMs of aged DKO mice, the levels of the iron importer DMT1 were investigated in these cells and found to be significantly decreased (*p* = 0.0251) (Figure 5a). In addition, the amount of the intracellular iron storage protein Ft was lower in aged DKO BMDMs compared to aged CTRLs (*p* = 0.0321) (Figure 5b). Collectively, these data show that a lack of Hfe andTfR2 in macrophages affected cellular iron import and export to different extents.

### 2.5. Lack of Hfe/TfR2 Affects Erythropoiesis in Aged DKO Mice

**Figure 6:**
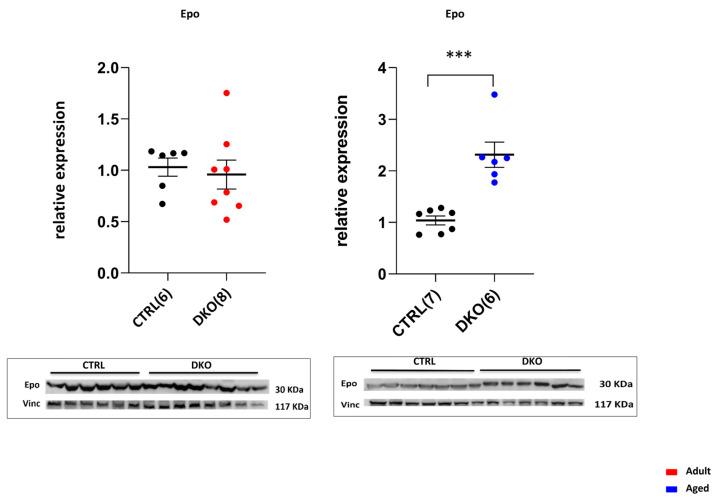
Kidney Epo production. Immunoblot of relative amounts of Epo in adult and aged DKO mice revealed a significant increase in aged DKO mice compared to CTRLs, while no differences were found in the adult DKO group. The number of analyzed animals is in parentheses. (*** *p* ≤ 0.001).

Erythropoietin (Epo) evaluation in DKO mice kidneys vs. CTRLs evidenced that aged DKO mice had significantly higher amounts of Epo compared to CTRLs (*p* = 0.0003), while no difference was found in adult DKO animals, demonstrating that there is an erythropoietic stimulus in aged targeted animals only (Figure 6). 

## 3. Discussion

This study reports the systemic and local effects of two major hemochromatosis proteins on iron metabolism in a new double knockout mouse model (Hfe/TfR2 KO, DKO), in which the *Hfe* and *TfR2* genes were selectively silenced in macrophages. 

It is known that Hfe and TfR2 cooperate in the modulation of the iron hormone Hepc in hepatocytes. Several studies on cell cultures indicate that Hfe and TfR2 interact as a complex that is required for Hepc activation [14,32], although the specific functional relationship between these two proteins is not clear. On the contrary, Schmidt et al. demonstrated that *Hfe* transgene-induced hepatic Hepc expression occurred even if *TfR2* is downregulated, suggesting that the interaction is not absolutely required for Hepc induction in vivo and that *TfR2* is not essential for the effects of the *Hfe* transgene on iron metabolism [33]. Furthermore, details on the possible role of Hfe and TfR2 in iron metabolism regulation in cells other than hepatocytes are not yet clear. The present study reports that silencing *Hfe* and *TfR2* in macrophages induced the upregulation of hepatic *Hepc* transcription, although it is dispensable for hepatic iron regulation, highlighting the macrophage-specific functions of these two Hereditary Hemochromatosis proteins [34,35] in the regulation of cellular and tissue iron homeostasis.

From a phenotypic point of view, DKO animals were viable and fertile, and no physical or behavioral abnormalities were observed compared to control mice at both analyzed ages, apart from a lower body weight in adult DKOs compared to age-matched controls. This could be due to the diminished iron provided by the adult DKO spleen, in which the iron exporter Fpn1 is reduced because of a hepatic Hepc increase. The iron available could be preferentially used for erythropoiesis, which, based upon the unaltered Erythropoietin (Epo) production in these mice, seems to be normal, at the expense of mice body growth.

The lower spleen weight without changes in their size usually indicates an impairment in splenic functionality, further confirming that adult DKO spleens present a lower intracellular iron amount.

**Figure 7:**
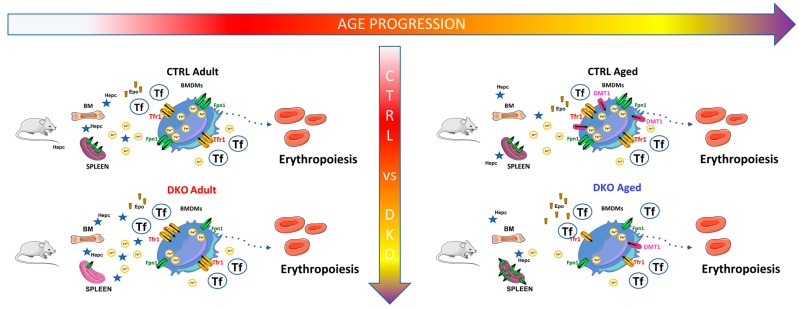
Proposed model on the effects of combined Hfe and TfR2 silencing in macrophages during aging. Left: adult DKO animals have splenic iron deficiency (light purple in the Figure), but no significant changes could be observed in serum iron parameters, irrespective of the lower amount of iron exporter Ferroportin 1 (Fpn1) due to an abnormally high Hepc production. In the animal’s bone marrow, targeted macrophages import more iron, increasing TfR1 so that erythropoiesis is maintained and Epo levels are comparable to those of aged-matched CTRLs. Right: in DKO aged animals, the spleen is not able to provide enough iron anymore to maintain erythropoiesis, and the serum iron parameters change according to an iron deficiency condition, which causes a reduction in Hepc. Concurrently, the iron intake of BMDMs is impaired through TfR1 and DMT1, an intracellular iron deficiency, and a decrease in Fpn1 amount, compromising the iron supply for erythropoiesis, whose impairment is manifested by an increase in Epo. BM: Bone Marrow; BMDM: Bone marrow-Derived Macrophages; DMT1: Divalent Metal Transporter 1; Epo: Erythropoietin; Fpn1: Ferroportin 1; Hepc: Hepcidin; Tf: Transferrin; TfR1: Transferrin Receptor 1.

The situation appears to be no longer compensated during aging so that, irrespective of the stop in animal growth, serum iron parameters significantly change in aged DKO mice, according to an iron deficiency condition demonstrated by a significant reduction in serum iron (SI) level, as well as lower Transferrin saturation, and a striking increase in sTf. In agreement, hepatic Hepc is reduced in aged DKO mice and splenic Fpn1 is consequently increased. All these data support the hypothesis of impaired erythropoiesis in these animals due to iron deficiency, confirmed by the increase in Epo production in the kidneys of aged DKO animals. Our results suggest that the combined silencing of *Hfe* and *TfR2* in macrophages leads to an alteration in the transcriptional regulation of splenic Fpn1 in adult DKO animals, which conflicts with the corresponding low protein amount. It would be interesting to clarify if this low Fpn1 amount is the consequence of the increased hepatic Hepc production rather than IRE/IRP-dependent post-transcriptional regulation [36]. As regards the splenic Fpn1 increase in DKO aged mice, it mirrored the Fpn1 detected in the spleens of single Hfe KO aged mice [30], but the additional lack of TfR2 causes an additional Fpn1 increase vs. Hfe single KO (2x vs 0.2, respectively). Nonetheless, splenic iron export by Fpn1 is not sufficient to satisfy the iron need in aged DKO mice, so serum iron deficiency develops in older animals. Again, the SI and Hepc amounts in DKO aged mice agree with the result obtained in the macrophage-specific Hfe KO mouse model of the same age [30], even if the DKO SI and Hepc levels are far lower than those of Hfe single KO, demonstrating that *TfR2* silencing further exacerbates the macrophages’ specific *Hfe* silencing effect on iron metabolism and confirming the role of Hfe and TfR2 in balancing serum iron levels in aged animals (Figure 7).

All these data strengthen the hypothesis on the cooperation of Hfe and TfR2 in iron metabolism outside of hepatocytes [37] and that the specific TfR2 loss of function causes the worsening of the altered macrophage cellular phenotype compared to Hfe KO [30]. 

Since both alpha and beta *TfR2* isoforms [38] are inactivated in the DKO animals, it is currently not possible to discriminate if one or both are involved in splenic Fpn1 regulation, but some speculations could be made comparing the results obtained on adult DKO spleens and TfR2 beta-specific knock out mice (TfR2 KI) of the same age [23]. While the spleens of adult KI mice were overloaded with iron, with decreased *Fpn1* transcription and translation, the adult DKO spleens are in the opposite situation. This could support the hypothesis of a functional competition between the TfR2 alpha and beta isoforms in the transcriptional regulation of Fpn1.

Considering that this novel mouse model presents macrophage-specific *Hfe* and *TfR2* deletion, iron metabolism in Bone Marrow-Derived Macrophages (BMDMs) was examined as they play a pivotal role in iron supply for erythropoiesis [27,28]. Here, it is shown that the targeted deletion of *Hfe* and *TfR2* resulted in reduced levels of the iron exporter Fpn1 in adult DKO animals. Similar findings were reported in TfR2-targeted peritoneal macrophages in a previous study [39], and it became particularly evident in aged DKO mice. While the *Fpn1* gene is transcriptionally downregulated in adult BMDMs, in aged mice, BMDMs were post-transcriptionally downmodulated by the IRE/IRPS system, which seemed to be functioning. In any case, a reduction in the level of the Fpn1 protein, confirmed in DKO mice through IF, usually occurs when intracellular iron levels are low. 

Iron deficiency in adult DKO BMDMs is corroborated by the results of TfR1, the primary iron importer, since the levels of this protein substantially increase. In contrast, TfR1 nearly disappears in the BMDMs of aged DKO mice, along with the iron importer DMT1. Along with TfR1, reduced levels of Fpn1, DMT1, and Ft suggest that the BMDMs of aged DKO mice have suppressed overall iron uptake and export compared to age-matched CTRLs.

It is undeniable that the data presented in this manuscript are mainly descriptive, although they could represent the starting point to finely clarify the molecular basis of the evident phenotypic alterations described here.

Moreover, the possibility of comparing the results from DKO BMDMs with the BMDMs of Hfe [30] and TfR2 [39] single macrophage-specific KOs allows us to draw some conclusions about the differential functions of the two proteins in macrophages. It appears clear that TfR2 controls Fpn1 gene transcription and protein production in BMDMs from adult mice, as they are decreased both in DKO and in TfR2 KO BMDMs [39], while Hfe is dispensable because no Fpn1 variation could be found in Hfe KO BMDMs from mice of the same age [30]. More complex is the situation regarding the BMDMs of aged mice. No data are available for aged TfR2 KO BMDMs, and although DKO and Hfe KO BMDMs are iron depleted, Fpn1 transcription is not modified and the protein is reduced in DKO, while it is increased in Hfe KO, along with the Fpn1 protein [30]. These data could be in favor of a predominant role of Hfe in regulating Fpn1 at this animal age. On the contrary, the TfR2-specific regulation of TfR1 expression in the BMDMs of aged mice is particularly evident and dominant over Hfe, since no changes in this protein could be found in Hfe KO BMDMs [30].

It is important to point out that the comparative analysis of inbred Hfe and Tfr2 single KOs in macrophages will significantly help in demonstrating the above-suggested hypothesis and they are worth studying in both adult and aged animals.

## 4. Materials and Methods

### 4.1. Mice

C57BL/6J/sv129 male mice of different ages (adult, 10 weeks old, and aged, 45–52 weeks old) were used. The animals’ age interval was chosen according to Harrison Laboratory’s online article (https://www.jax.org/research-and-faculty/research-labs/the-harrison-lab/gerontology/life-span-as-a-biomarker (accessed on 30 July 2024)). Each group of mice was fed with a standard diet (VRF1, Special Diets Services, Essex, United Kingdom). Animal housing and all the experimental procedures were performed in accordance with European (Official Journal of the European Union L276, 20/10/2010, Vol. 53, pp. 33–80) and National Legislation (Official Journal n° 61, 14 March 2014, pp. 2–68) for the protection of animals used for scientific purposes, and the experimental procedure was approved by the Ethical Committee of the University of Turin and conducted according to the ARRIVE guidelines. 

The number of mice analyzed in each experiment varied on the basis of the necessity of performing multiple analyses on small-sized organs and the small amount of material obtained (in the case of BM-derived macrophages), as well as the physiological time needed for animal aging, taking in consideration the 3Rs principle for animal experimentation. The higher number of 9 animals was utilized to measure noninvasive parameters.

Mice were anesthetized before all invasive procedures with ketamine, 100 mg/kg (Ketavet, Bayern, Leverkusen, Germany; Xylazine, 5 mg/kg; Bayer, Milan, Italy), and sacrificed by cervical dislocation.

### 4.2. DNA Extraction and Genotyping 

A mouse tail biopsy was treated with 600 μL of lysis buffer (100 nM Tris-Cl pH 8, 5 mM EDTA pH8, 200 mM NaCl, 0.2%SDS) and 10 μL of proteinase K (Promega S.r.l, Milan, Italy), and incubated at 55 °C for 3 h. DNA extraction was achieved using a phenol/chloroform-based procedure. The DNA pellet was resuspended in 200 μL of dH2O. PCR reaction for genotyping was performed with primers and protocols specific for the Hfe, TfR2, and Cre-recombinase genes (Supplemental Table S1).

### 4.3. Serum Iron

Blood was collected from the retro-orbital sinus of anaesthetized mice. Blood samples were centrifuged at 1500 rpm for 10 min to collect serum. Serum iron (SI) levels were assessed using a commercial kit (Iron Direct Method, Bioabo, Mainz, France), following the manufacturer’s instructions. TS was measured using the ab239715 Kit (Abcam Ltd., Cambridge, UK) according to the manufacturer’s suggestions. The absorbance was measured in a spectrophotometer at a wavelength of 600 nm. 

### 4.4. Liver and Spleen Non-Heme Iron Content 

Following dissection, part of the tissues were taken to analyze liver and spleen nonheme iron content (LIC and SIC, respectively). This analysis was performed according to the standard reported protocol [23].

### 4.5. Prussian Blue Perls’ Staining

After sacrifice and dissection, the liver and spleen were explanted, post-fixed in PFA for 24 h at 4 °C, and cryoprotected in 30% sucrose in 0.12 M phosphate buffer. Tissues were cut by cryostat in 30 μm thick sections and were stained for non-heme ferrous iron by Prussian blue Perls using a commercial kit (Bio-Optica, Milan, Italy).

### 4.6. Isolation of Bone Marrow-Derived Macrophages (BMDMs)

The animal’s femur and tibia were transferred into a cell culture plate with 80% ethanol for 15 s to decontaminate them and then in DMEM (Sigma, Steinheim, Germany) to remove excess ethanol. Then, both ends of the bones were cut and the bone marrow was flushed with 2.5 mL of medium. Cells were filtered and the suspension was centrifuged at 1500 rpm for 7 min at 4 °C. The supernatant was discarded and the pellet was suspended in a culture medium containing DMEM with 400 mg/L glucose, 100 mM L-glutamine (Invitrogen, Waltham, MA, USA), 100 mM sodium pyruvate (Sigma, Steinheim, Germany), penicillin/streptomycin 100x (Sigma, Steinheim, Germany), 10x FBS (Sigma, Steinheim, Germany), and the culture supernatant of L929 cells, obtained via the standard culture protocol [40]. The suspended pellet was divided into three 100 mm Petri dishes (Aurogene, Rome, Italy) for 10 days of incubation at 37 °C, 5% CO_2_. The DMEM medium was changed every two days. After ten days of culture, the differentiated macrophages were collected and stored at −80 °C.

### 4.7. Real-Time Quantitative PCR

Total RNA was isolated from homogenized livers, spleens, and bone marrow cellular pellets using Trizol Reagent (Ambion, Thermo Fisher Scientific, Waltham, MA, USA), according to the manufacturer’s protocol. Reverse transcription was performed with an iScript kit, (Bio-Rad, Hercules, CA, USA), following the manufacturer’s recommendation of 1–3 μg of RNA. The expressions of Hepc, Fpn1, Hfe, DMT1, and β-glucuronidase (Gus-β) were evaluated with TaqMan technology (TaqMan Universal Master Mix, Thermo Fisher Scientific, Waltham, MA, USA). The AoD codes of each gene are reported in Table S2. TfR1 and TfR2 expressions were determined using CYBR Technology; each gene-specific primer is reported in Table S2. The C1000 Thermal Cycler CFX96 Real-Time System (Bio-Rad, Hercules, CA, USA) was utilized. All analyses were carried out in biological and technical replicates and the target mRNA was compared to age-matched DKO and CTRL mice groups. Transcriptional data were evaluated by taking 2^-delta delta Ct values normalized to the mean of age-matched CTRLs.

### 4.8. Protein Isolation 

Livers, spleens, kidneys, and BMDMs were lysed using the RIPA lysis buffer (150 mM NaCl, 1% NP-40, 0.5% Sodium Deoxycholate, 0.1% SDS 50 mM Tris-Cl pH 8.0), together with a mixture of protease inhibitors (Santa Cruz Biotechnology, Dallas, TX, USA), and the lysate was incubated for 30 min in a shaker at 4 °C. The samples were centrifuged for 15 min at 13,000 rpm at 4 °C and the supernatant was collected. The quantification with the colorimetric method (Protein Assay Dye reagent, Bio-Rad) was carried out at a final dilution of 1:1000.

### 4.9. Western Blotting (WB)

An average amount of 30 μg of protein was separated by SDS-PAGE electrophoresis with a 6–12% acrylamide gel, while 2 μL of serum was used for Transferrin determination. Electrophoresis was performed and the proteins were transferred to nitrocellulose membranes (GE Healthcare Life Sciences, Milan, Italy). The membranes were incubated overnight at 4 °C with primary antibodies against anti-Fpn1, DMT1, FtH, Tf, and β−actin, all provided by Santa Cruz Biotechnology, USA, as well as anti-Epo (Immunological Sciences, Rome, Italy) and anti-TfR1 and Vinculin antibodies (Invitrogen, Waltham, MA, USA). Following incubation with the secondary antibodies for 1 h at RT, immunoreactivity was detected using the chemiluminescence kit (Western Lightning Plus ECL, Newton Abbot, UK) and a ChemiDoc XRS instrument. For protein quantification, Image Lab 4.0.1 software (Bio-Rad, Hercules, CA, USA) was used.

### 4.10. Immunofluorescence (IF)

Macrophages isolated from bone marrow (BMDMs) were collected and plated in culture in 6-well plates with slides for 1–2 days to allow engraftment. The slides were then washed with PBS and fixed with 4% paraformaldehyde (PFA) in PBS pH 7.4 for 10 min at RT. The cells were then treated with 3% blocking solution in 1x PBS for 30 min, then primary antibody anti-Fpn1 (see above) was added to the cell suspension, which was stored overnight at 4 °C. After washing, the cells were incubated with the secondary antibody Alexa fluor 488 Green (1: 500, Invitrogen, Waltham, MA, USA) for 1 h at RT in the dark. Then, 1 µL of DAPI (4,6-diamidino-2-phenylindole) was added for 5 min to stain nuclei and subsequently washed with dH2O. The slides were dried and mounted on microscope slides with tris-glycerol supplemented with 10% Moviol (Calbiochem, LaJolla, CA, USA) and examined with the Zeiss LSM 800 confocal laser scanning microscope (Zeiss, Feldbach, Switzerland). The images were taken at 63x magnification. Signal strength was measured using ImageJ 1.38v software. BMDMs from 3 different animals for each genotype/age were analyzed. Fluorescence from thirty-five cells have been evaluated for each mouse. The Fpn1 fluorescence was normalized to nuclei (DAPI).

### 4.11. Statistical Analysis

Statistical analyses were performed using GraphPad Prism 7.00 software, in which DKO mice were always compared to age-matched CTRL animals. All the graphs show error bars that denote standard deviation, and the values are normalized using the mean of the CTRL animals. For statistical analysis, an Unpaired Student *t*-test (two-tailed) was used. All analyses with *p* < 0.05 were indicated as statistically significant (* *p* ≤ 0.05; ** *p* ≤ 0.01; *** *p* ≤ 0.001).

## Data Availability

Data is contained within the article and Supplementary Saterial.

## Supplementary Materials

The supporting information can be downloaded at: https://www.mdpi.com/article/10.3390/ijms25179142/s1.
