# Supplementary material for: Lack of Hfe and TfR2 in Macrophages Impairs Iron Metabolism in the Spleen and the Bone Marrow
Source: Int J Mol Sci. 2024 Aug 23;25(17):9142. doi: 10.3390/ijms25179142 (PMC11395440; doi:10.3390/ijms25179142)
Supplement: Supplementary file 1 [file ijms-25-09142-s001.zip › ijms-3126672-supplementary.pdf]

Comità et al., Supplementary Materials

Table S1

PCR conditions for DKO mice genotyping

GENE	MIX	FORWARD PRIMER	REVERSE PRIMER	PROTOCOL
**Hfe**	1X buffer; primers 10 µM; DNA Polymerase 2,5 U; DNA 20-50 ng	5’-CACAGTAAGGGTACGTGGAG-3’	R1: 5’-TGGAGACAGTGCAGTAGGC-3’<br>R2: 5’-AGGGTCACAAACAGCCATAC-3’	1x5 min at 95°C, 30x1 min at 95°C, 1x30’’ at 63°C, 10 min at 72°C.
**TfR2**		5’-CATGGCTTGTCCAAATGCT-3’	5’-CCAGCTTCTTAAACCATA-3’	1x 2 min at 94°C, 35x 30’’ at 94°C, 30’’ at 58°C, 30’’ at 72°C
**Cre**		5’-AGGCGTTTCTGAGCATACC-3’	5’-TAGCTGGCTGGTGGCAGATG-3’	1x2 min at 94°C, 32x 20 sec at 94°C, 20’’ at 61°C, 20’’ at 72°C

Table S2

GENE	METHOD	PROBES	PRIMER SEQUENCES
**Hepcidin (Hepc)**	Assay on Demand	Mm00519025_m1	/
**Fpn1 (SLC40A1)**	Assay on Demand	Mm00446953_m1	/
**Hfe**	Assay on Demand	Mm00439314_m1	/
**DMT1**	Assay on Demand	Mm00435354_m1	/
**Gus-β**	Assay on Demand	Mm99999915_g1	/
**TfR2**	CYBR Green	/	Forward primer: 5’CCTGGCCCCTAGTGTGATTTC3’<br>Reverse primer: 5’TGGCGCGAGAGCTTATCG3’
**TfR1**	CYBR Green	/	Forward primer: 5’GTAGCAGCTGAGAATGATGG3’<br>Reverse primer: 5’AGCCAGTTTCATCTCCACATG3’
**Gus-β**	CYBR Green	/	Forward primer: 5’GGGACCATCGTCTACAAGACTGA3’<br>Reverse primer: 5’GCTTGTGTCCTGGACAAAGTAACC3’

Figure S1

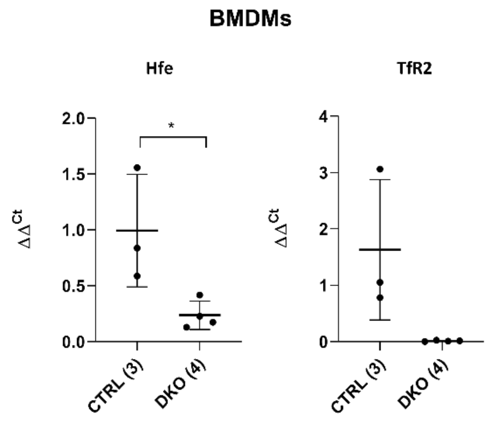

Figure S1. Hfe and TfR2 genes expression in DKO BMDM compared to CRTL in adult mice. The transcriptional levels of the two genes were performed by quantitative real-time PCR analysis. Gus-β was utilized as internal control. 2^-delta Ct was used for analyses and the number of analysed animals is in parenthesis (* $p \leq 0.05$).

Figure S2

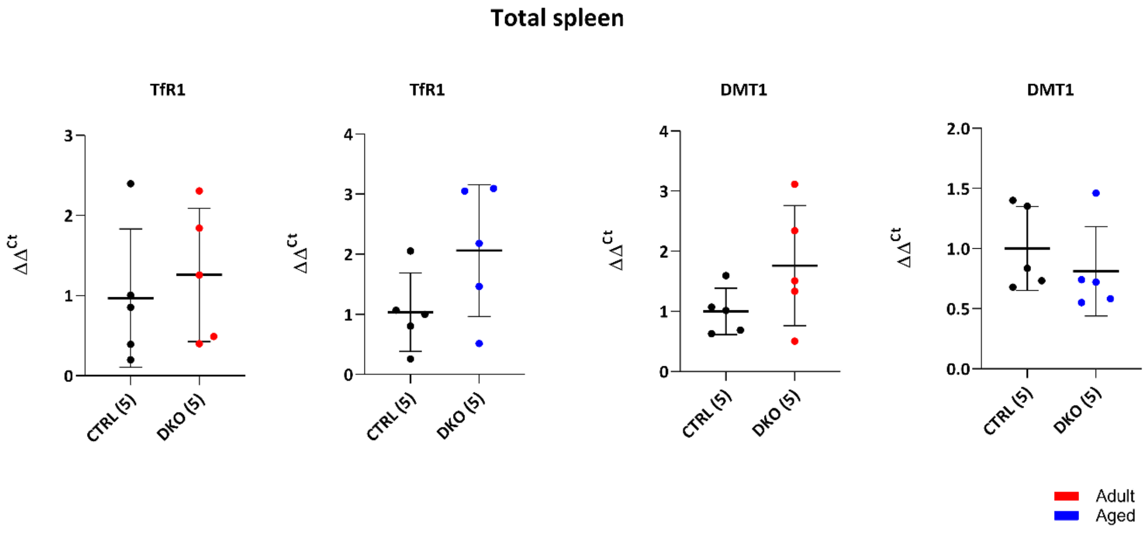

Figure S2. Splenic TfR1 and DMT1 transcriptional levels in CTRL and DKO adult/aged mice. Graphs representing TfR1 and DMT1 mRNA expression. The transcriptional levels of the two genes were performed by quantitative real-time PCR analysis. Gus-β was utilized as internal control. 2^-delta Ct was used for analyses. The number of analysed animals is in parenthesis.
